# Left Atrial Appendage Closure in Patients with Atrial Fibrillation and Intermediate-to-Borderline High Cardiovascular Risk: A Retrospective Propensity Match Cohort Study

**DOI:** 10.3390/jcdd13010041

**Published:** 2026-01-11

**Authors:** Jiayi Liu, Ningjing Qian, Ying Gao, Junyan Jin, Bingqi Wang, Muhua Luo, Yaping Wang

**Affiliations:** 1 Department of Cardiology, The Second Affiliated Hospital, School of Medicine, Zhejiang University, Hangzhou 310009, China; 22318378@zju.edu.cn (J.L.); njqian@zju.edu.cn (N.Q.); 12218232@zju.edu.cn (Y.G.); junyanjin@zju.edu.cn (J.J.); 22418359@zju.edu.cn (B.W.); 22418422@zju.edu.cn (M.L.); 2 State Key Laboratory of Transvascular Implantation Devices, Hangzhou 310009, China; 3 Heart Regeneration and Repair Key Laboratory of Zhejiang Province, Hangzhou 310009, China

**Keywords:** atrial fibrillation, left atrial appendage closure, oral anticoagulation, cardioembolic event, stroke prevention

## Abstract

Background and objective: Evidence of percutaneous left atrial appendage closure (LAAC) and oral anticoagulants (OACs) in non-valvular atrial fibrillation (NVAF) patients with intermediate-to-borderline high stroke risk is scarce. We aimed to compare the efficacy and safety of these treatments in the latter clinical population. Methods: This retrospective cohort study included NVAF patients with CHA_2_DS_2_-VA scores of 1–2 and used 1:1 propensity score matching (184 patients per group) to compare efficacy and safety outcomes. The primary efficacy outcome was a composite of stroke, transient ischemic attacks, systemic embolism, and cardiovascular death during follow-up. Adverse safety events were categorized into peri-procedure (LAAC group) and non-procedural (both groups) events. Results: Over a mean follow-up of 48.93 ± 28.50 months, a total of 26 patients (7.07%) reached the primary composite efficacy endpoint. The LAAC group showed a significantly higher incidence of the efficacy endpoint compared to the OAC group (HR = 3.09; 95% CI 1.22–7.85; log-rank *p* = 0.01). Procedure-related events occurred in five LAAC patients (one contributing to primary endpoint), while non-procedural bleeding rates were similar (0.54% vs. 1.09%; *p* = 0.56). Subgroup analyses suggested concomitant ablation of NVAF in LAAC group did not significantly improve efficacy composite endpoints (HR = 0.47). Conclusions: In NVAF patients with intermediate-to-high stroke risk, OACs were more effective than LAAC in preventing thromboembolic events, with comparable rates of clinically relevant bleeding.

## 1. Introduction

Atrial fibrillation (AF) affects over 50 million individuals globally [1], with an annual incidence of 4.48 million cases, resulting in approximately 340,000 deaths and 8.36 million disability-adjusted life-years [2]. As the underlying cause of 25% of ischemic strokes [3], non-valvular AF independently increases stroke risk fivefold and mortality risk twofold [4]. While the CHA_2_DS_2_-VASc score remains the primary tool for stroke risk stratification [5], contemporary evidence from large cohorts demonstrates no significant association between female sex and thromboembolic risk after multivariable adjustment, supporting the comparable predictive validity of the CHA_2_DS_2_-VA score [6,7].

Current therapeutic strategies are well-established. Direct oral anticoagulants (DOACs), such as dabigatran, rivaroxaban, apixaban, and edoxaban, have emerged as first-line therapy, demonstrating superior safety profiles to warfarin [8].

For patients with contraindications to long-term anticoagulation, left atrial appendage closure (LAAC)—an FDA-approved transcatheter procedure that occludes the primary thrombogenic site in non-valvular AF—represents the most extensively studied alternative for stroke prevention [9]. Several randomized controlled clinical trial (RCT) studies have demonstrated that LAAC is non-inferior to warfarin in stroke prevention with reduced bleeding risk [10,11]. However, the current guidelines primarily support its application in high-risk patients who are not candidates for long-term anticoagulation. Evidence remains limited for intermediate-risk patients (i.e., CHA_2_DS_2_-VA score = 1). Even among borderline high-risk patients (CHA_2_DS_2_-VA score = 2), there is notable heterogeneity in stroke rates, with significantly lower thromboembolic risks compared to patients with scores of ≥3 [12].

Herein, we reported on a retrospective propensity-matched cohort study addressing therapeutic uncertainty in intermediate-to-borderline high-stroke-risk NVAF patients by directly comparing LAAC with oral anticoagulation, which evaluates both efficacy and safety outcomes to inform clinical practice.

## 2. Methods

### 2.1. Study Design and Criteria for Eligibility

This retrospective cohort study was conducted at the Second Affiliated Hospital of Zhejiang University School of Medicine, China, including hospitalized NVAF patients admitted from 1 April 2014 to 1 April 2024. The baseline data of eligible participants and follow-up information were retrieved from the hospital’s electronic medical record system. Follow-up started from hospital admission and ended on the date of efficacy outcome occurrence or 31 August 2024. The study protocol complied with the principles of the Declaration of Helsinki and was approved by the Ethics Committee of the Second Affiliated Hospital of Zhejiang University School of Medicine (2024-0348). Due to the retrospective nature of this study, the ethics committee waived the need for written informed consent from the patients enrolled, besides that of the interventional procedure.

The inclusion criteria were as follows: (1) patients aged 18 years and older with a diagnosis of NVAF who have a CHA_2_DS_2_-VA score of 1–2 and the (2) ability to communicate well with the investigator and adhere to the requirements of the entire trial. The exclusion criteria were as follows: (1) the presence of malignant tumors or severe hematologic disorders; (2) valvular atrial fibrillation, severe congenital heart disease, or cardiac amyloidosis; (3) contraindications to OACs; (4) comorbidities necessitating prolonged anticoagulation independent of atrial fibrillation; (5) end-stage chronic hepatic disease or severe renal impairment (e.g., CrCl < 15 mL/min); (6) presence of thrombus in the left auricle; (7) hyperthyroidism; (8) diffuse connective tissue diseases; or (9) significant missing baseline data.

### 2.2. Oral Anticoagulation and LAAC Procedures

The allocation of patients to LAAC or OAC therapy was based on comprehensive clinical considerations and shared decision-making. LAAC was typically indicated for patients with high bleeding risk (HAS-BLED score ≥ 3 or history of major bleeding), contraindications or intolerance to long-term anticoagulation, strong patient preference for device-based therapy after detailed counseling, or complex LAA anatomy unsuitable for long-term anticoagulation. The decision was made through multidisciplinary team discussion involving cardiologists, electrophysiologists, and neurologists, taking into account individual patient characteristics, comorbidities, and preferences. For patients with low-to-moderate bleeding risk and no contraindications to anticoagulation, OAC therapy was the preferred approach.

Oral anticoagulation: Patients in the OAC group received either warfarin (target INR 2.0–3.0) or once standard-dose DOAC, including dabigatran (150 mg twice daily), rivaroxaban (20 mg once daily), apixaban (5 mg twice daily), or edoxaban (60 mg once daily) [5]. DOACs were prioritized due to their favorable bleeding profile, while warfarin was reserved for patients with contraindications to DOACs, such as those with severe renal impairment (CrCl 15–30 mL/min).

LAAC procedures: Pre-procedural assessment encompassed laboratory testing, electrocardiography, and transthoracic echocardiography. Furthermore, left atrial computed tomography angiography (CTA) or transesophageal echocardiography (TEE) was essential for an anatomical evaluation of the left atrial appendage and exclusion of intracardiac thrombus.

The procedure was performed under local anesthesia with moderate-to-deep intravenous sedation. After coronary sinus catheter placement via femoral access, TEE-guided transseptal puncture was performed. Intra-procedural multi-angle measurements and triple-view angiography were used to assess optimal device positioning. Once the COST criteria (circumflex artery, opening, sealing, and tug test) were confirmed, an appropriately sized LAA occluder was deployed. Available device options included the WATCHMAN 2.5, LAmbre, SeaLA, LAmax, and Leftear. Following LAAC, patients routinely receive either anticoagulation monotherapy (standard-dose DOAC or warfarin with INR 2–3) or anticoagulation with SAPT therapy (anticoagulation plus 100 mg aspirin daily for patients with a history of coronary artery disease, ischemic stroke, or peripheral arterial disease, who are at moderate to high thrombotic risk) for at least 6 weeks. A follow-up TEE or left atrial CTA is typically performed at 45 days post-procedure to assess device positioning and potential transition to antiplatelet therapy.

The decision for combined RFCA and LAAC therapy was made on a case-by-case basis, with some patients voluntarily opting for this approach after thorough counseling. For patients undergoing concomitant procedures, the intervention followed an “ablate-first, close-later” strategy. The procedure began with right femoral venous access to place coronary sinus and right ventricular electrodes. After transseptal puncture, bilateral pulmonary vein angiography and left/right heart catheterization were performed. A Pentaray catheter was used to create 3D electroanatomical maps of the left atrium and LAA. Based on the mapping findings, ablation was performed at 30–50 W, including standard pulmonary vein isolation with additional linear or fragmented signal ablation as needed. Sinus rhythm was restored either by ablation or electrical cardioversion. LAAC was then performed followed by the anticoagulation regimen as described above.

### 2.3. Outcome Measure

The primary efficacy outcome was a composite endpoint consisting of TE events (ischemic stroke, hemorrhagic stroke, TIA, and SE) and cardiovascular death. Safety adverse events were categorized as peri-procedure adverse events of the LAAC group and non-procedural-related adverse events of both groups. Peri-procedure adverse events were defined as events occurring within 7 days post-LAAC, including perioperative death, device migration or dislodgement, device-related embolization, pericardial effusion requiring surgical or percutaneous transluminal drainage, groin and retroperitoneal hematoma formed, and procedure-related stroke. Non-procedural-related adverse events mainly focused on clinical-relevant major bleeding, which is defined as a drop in hemoglobin of 2 g/dL or the need for a transfusion of 2 units of filled red blood cells or whole blood [13], and some other OAC-related side effects (nausea, vomiting, diarrhea, rash, liver function abnormalities, and headache).

### 2.4. Statistical Analysis

The demographics and clinical characteristics between the two groups were compared by a chi-square test (for categorical variables), independent samples t-test, or Mann–Whitney U test (for continuous variables), as appropriate. To mitigate the covariate imbalance, we used demographic characteristic variables, laboratory tests, scoring data, and comorbidities that were statistically significantly different and matched the 1:1 propensity score using the nearest neighbor matching method with a caliper value of 0.25.

We used Kaplan–Meier survival analysis and the log-rank test to estimate the cumulative hazard and assess time-to-event outcomes for patients in the OAC and LAAO groups. Cox proportional hazard models were used for quantifying the relative risk of events among groups. In addition, we also performed a subgroup analysis using a forest plot to visualize interaction analyses by COX regression. Interaction *p*-values were reported to identify statistically significant differences in treatment effects.

Statistical analyses for this article were implemented using SPSS 25.0 (SPSS Software Inc., Chicago, IL, USA), R version 4.3.0 (R Project for Statistical Computing). *p* < 0.05 was considered statistically significant.

## 3. Results

### 3.1. Patient Characteristics and Propensity Score Matching

**Figure 1:**
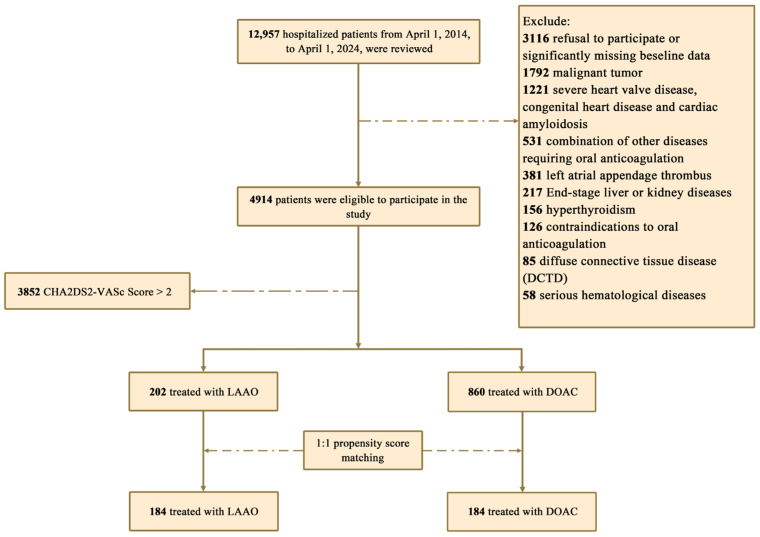
Flow chart.

A total of 12,597 patients were initially screened from the electronic medical record system, with 4914 patients meeting inclusion criteria. In this retrospective cohort, 1062 patients (LAAC = 202; OAC = 860) had a CHA_2_DS_2_-VA score of 1–2 (Figure 1). Baseline data revealed significant differences between groups prior to matching, with the LAAC group having a larger proportion of patients > 75 years (5.45% vs. 1.51%), high bleeding risk (10.90% vs. 3.26%), and a higher mean CHA_2_DS_2_-VA score (1.63 ± 0.48 vs. 1.32 ± 0.46). After 1:1 propensity score matching, the final cohort comprised 368 patients (184 per group), with most covariates achieving standardized mean differences (SMDs) < 0.2, indicating that intergroup covariate imbalance was ameliorated (Table 1). The mean age was 62 ± 8 years, and matching resulted in similar mean CHA_2_DS_2_-VA scores (1.61 ± 0.49 vs. 1.53 ± 0.50) and mean HAS- BLED scores (1.38 ± 0.89 vs. 1.26 ± 0.88).

In the OAC group, 64 patients (34.78%) received warfarin therapy with a mean INR of 2.05 ± 0.64 during treatment. The remaining patients were prescribed standard-dose DOACs (dabigatran: 17 patients (9.24%); rivaroxaban: 96 patients (52.17%); edoxaban: 7 patients (3.81%)). In the LAAC group, 107 patients (58.70%) were implanted with WATCHMAN 2.5, 36 patients (19.57%) with LAmbre, 28 patients (15.22%) with LAmax, and 13 patients (6.51%) with other occluders. Complete left atrial appendage occlusion was successfully achieved in 179 patients (97.3%), with immediate peri-device leakage observed in four patients (2.2%), including three patients with ≤3 mm leaks and one patient with >3 mm leakage. Post-procedural therapy comprised anticoagulation monotherapy in 177 patients (96.2%) and anticoagulation with single-antiplatelet therapy (6-week course) in 7 patients (3.8%). Follow-up imaging assessment at 6 weeks revealed persistent peri-device leak > 3 mm existing in two patients (1.1%), both of which showed complete sealing at 6 months post-procedure.

### 3.2. Oral Anticoagulants Outperform LAAC in Stroke Prevention Without Increasing Major Bleeding Risk in Moderate-to-Borderline High-Stroke-Risk NVAF Patients

The mean follow-up time in this study was 48.93 ± 28.50 months, with no significant difference in follow-up time between the two groups (LAAC 48.36 ± 34.14 vs. OAC 49.49 ± 21.52).

**Figure 2:**
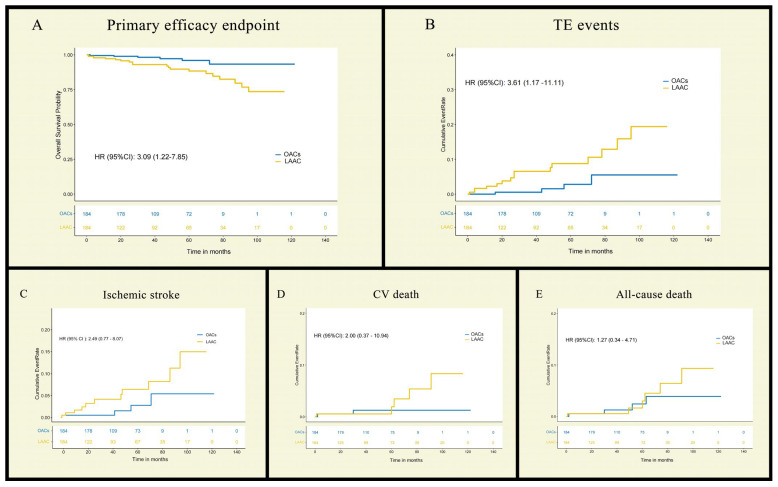
Kaplan–Meier curve and cumulative incidence curve of efficacy endpoints. Primary efficacy endpoint (**A**) includes stroke, TIA, SE, and cardiovascular death, in which former three constitute TE events (**B**). In addition, (**C**–**E**) show cumulative incidence curve of ischemic stroke, cardiovascular death, and all-cause death, separately. SE: systemic embolism; TIA: transient ischemic attack; TE: thromboembolic.

**Figure 3:**
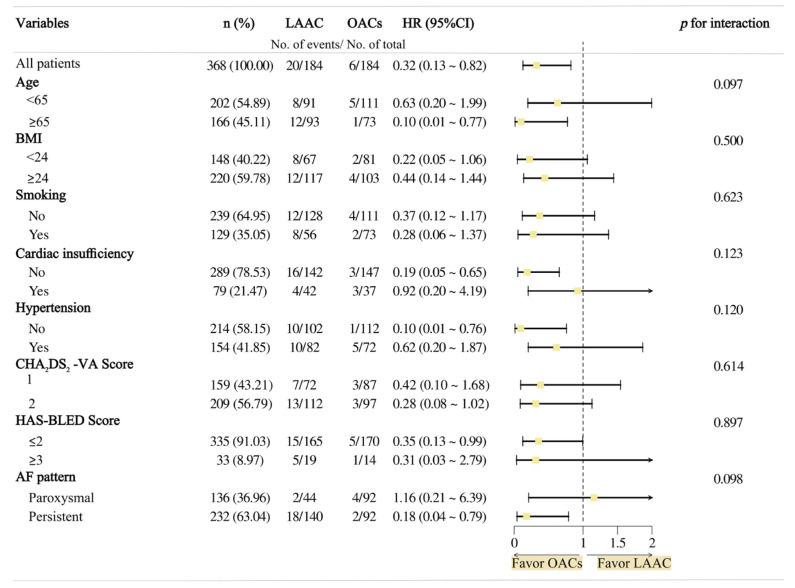
Subgroup analysis of primary efficacy endpoint (OACs versus LAAC).

During the follow-up period, 26 patients (7.07%) reached the composite efficacy endpoint (TE and CV death), with significantly higher incidence in the LAAC group (20 vs. 6 events; HR 3.09; 95% CI 1.22–7.85; log-rank *p* = 0.01; Rate/1000 person-months 219.78 vs. 49.18) (Table 2 and Figure 2). Of all 20 patients who reached efficacy endpoints in the LAAC group, half (n = 10) received the WATCHMAN 2.5 device, while the other half (n = 10) were implanted with other brands, indicating no significant influence of device type on clinical outcomes. When the treatments and the confounding variables mentioned above were included in the multivariate COX proportional risk model by non-collinearity tests, the effect of LAAC or OACs on the composite efficacy endpoint was still significant (HR = 3.43; 95% CI 1.15 ~ 10.21; *p* = 0.03). In the subgroup analyses, this result was consistent across all except for the pattern of AF, but the interaction *p*-value was greater than 0.05 (Table 3 and Figure 3). Among these, 15 cases were stroke events, all of which were ischemic strokes—11 in the LAAC group and 4 in the OAC group—with no statistically significant difference between the two groups (HR = 2.49; 95% CI 0.77–8.07; log-rank *p =* 0.12). And taking 30 days post-intervention as the time point, TE events occurred early in the postoperative period in only 2 patients (1.09%), with those in the remaining 13 (7.07%) occurring at a much longer time to surgery. In the LAAC group, there were two cases each of TIA and SE (systemic embolism), whereas no such events were observed in the OAC group. Furthermore, no significant differences were observed between the groups regarding CV death (LAAO 5 vs. OACs 2; HR = 2.00; 95% CI 0.37–10.94; log–rank *p* 0.41). In terms of non-cardiovascular mortality, there were three cases in total. In the LAAC group, one patient succumbed to severe COVID-19 infection, while in the OAC group, two patients died—one due to pancreatic cancer and the other from lymphoma.

Regarding adverse events, in the LAAC group, four patients (2.17%) experienced major procedure-related complications. One patient (0.54%) suffered device embolization, resulting in the dissection of the abdominal aorta, while the other three (1.53%) presented with large effusions requiring surgical intervention or percutaneous pericardiocentesis [14]. During postoperative follow-up, there was no significant difference in the incidence of late non-procedure-related clinically relevant bleeding between the LAAC and OAC groups (LAAC 1 vs. OACs 2, HR (95% CI) 0.50 [0.05–5.54], log-rank *p* = 0.56, Rate/1000 person-months 10.99 vs. 16.39). In the OAC group, 64 patients (34.78%) were on warfarin therapy, with 1 patient (0.54%) experiencing clinical gastrointestinal bleeding. Additionally, four patients (2.17%) developed hepatic dysfunction during anticoagulation, and two patients (1.09%) experienced minor bleeding events that did not require medical intervention. The remaining 120 patients (65.22%) received DOACs, including rivaroxaban, dabigatran, and apixaban. Among these, 1 patient (0.54%) experienced clinically intra-articular bleeding, while 10 patients (5.43%) had minor gingival, nasal, or mucocutaneous bleeding that resolved with the temporary discontinuation or switching of the medication. In addition, two patients reported gastrointestinal symptoms, and two experienced post-administration headaches (Table 4).

### 3.3. Concomitant Ablation of AF in LAAC Group Did Not Significantly Improve Efficacy Composite Endpoints

In the LAAC group, a total of 134 patients (72.83%) received concomitant LAAC and RFCA for AF. Among 73 patients (39.67%) who underwent circumferential pulmonary vein isolation (CPVI), 61 cases received additional linear ablation based on substrate modification during the procedure. The distribution of additional linear ablation was as follows: roof line in 33 cases (17.93%), anterior wall line in 14 cases (7.61%), and posterior wall box line in 14 cases (7.61%).

**Figure 4:**
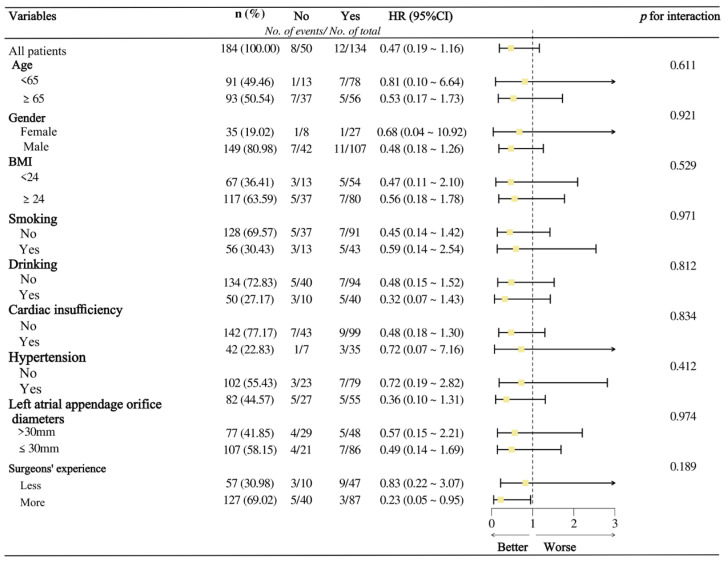
Subgroup analysis of concomitant ablation of AF and surgeons’ experience in LAAC groups.

There was no statistically significant difference in the incidence of composite efficacy endpoint events between the LAAC group and the combined AF ablation group (8.96% vs. 16.00%, *p* = 0.21). This directional consistency was maintained across subgroups stratified by surgeons’ experience (all *p*-interaction > 0.05) (Figure 4).

## 4. Discussion

Patients with NVAF face a substantially elevated risk of thromboembolic stroke, with approximately 90% of thromboembolic (TE) events originating from left atrial thrombi, particularly within the left atrial appendage [15]. LAAC is recognized as an alternative to OACs for stroke prevention in AF patients at high risk. It is recommended as a Class I therapy (Level B evidence) for patients with AF undergoing cardiac surgery. However, its application in non-surgical patients is more restricted, classified as a Class IIb recommendation (Level C evidence), primarily for individuals with contraindications to long-term anticoagulation or as an alternative preferred by the patient after comprehensive risk–benefit discussion [5,16]. The Norwegian AFNOR study [17], conducted among NVAF patients at intermediate stroke risk (CHA_2_DS_2_-VA = 1), demonstrated that non-anticoagulated patients exhibited a higher risk of stroke compared to those receiving anticoagulation therapy, while the incidence of intracranial hemorrhage associated with anticoagulation treatment was generally low. However, current guidelines remain inconclusive regarding anticoagulation therapy for intermediate-risk patients, creating significant challenges in clinical decision-making. Given this discrepancy between clinical benefits and guideline recommendations, the evaluation of anticoagulation treatment outcomes in this population becomes imperative.

Our study was conducted in a matched population with AF at intermediate-to-borderline high stroke risk (CHA_2_DS_2_-VA score 1–2) to provide guidance for their unspecified treatment, and the main findings of this study are as follows:

(1) OACs were associated with a significantly lower incidence of the primary composite efficacy endpoint (TIA, TE, and cardiovascular death) compared to LAAC.
(2) The incidence of non-procedural clinically relevant bleeding (defined as ISTH major bleeding) was similar between the OAC and LAAC groups.

OACs provide systemic protection against thromboembolic events, offering distinct advantages over LAAC, which primarily targets cardiac thrombus formation. Approximately 10% of strokes originate from non-cardiac sources, underscoring the importance of the comprehensive protection afforded by OACs. Previous studies have demonstrated that warfarin reduces the risk of stroke by approximately 60% compared to placebo, while DOACs provide additional benefits, including a further 19% reduction in stroke or systemic embolism and a 10% reduction in mortality [18]. Importantly, extensive data indicate that OACs not only reduce the frequency but also mitigate the severity of ischemic stroke when maintained at therapeutic levels [19]. However, in clinical practice, many patients remain hesitant about OAC therapy, with 40% of indicated patients declining anticoagulation therapy and 10–20% having absolute or relative contraindications [20]. Adherence remains another significant barrier, with 21–33% of patients discontinuing treatment within 24 months [21,22,23], primarily due to concerns about chronic renal failure and major bleeding. Consequently, LAAC emerges as an alternative strategy for preventing thromboembolic events in patients who are unable to adhere to or tolerate long-term anticoagulation therapy.

Several prior RCTs and registry studies have compared the efficacy and safety of LAAC and OAC in NVAF patients. At a mean follow-up of 18 months, the PROTECT AF trial demonstrated that LAAC achieved non-inferiority relative to warfarin for the composite efficacy endpoint. However, consistent with our findings, as shown in a subgroup analysis of AF patients with a CHADS_2_ score of 1, comprising 32% of the cohort, the composite endpoint failed to achieve non-inferiority. Moreover, the intervention group had a higher incidence of ischemic stroke than the control group [11]. In the PREVAIL trial, which included 6.63% of patients with a CHA_2_DS_2_-VA score of 1–2 and followed them for a similar duration, the relative risk (RR) for the composite efficacy endpoint was 1.07 (95% CI: 0.57 to 1.89), failing to meet the predefined non-inferiority margin of 1.75. Unfortunately, the study did not conduct a subgroup analysis of the 27 patients with efficacy events, limiting the interpretation of the findings in this cohort [10]. However, in our retrospective study, LAAC demonstrated better outcomes in the composite endpoint compared to OAC, despite a higher incidence of ischemic stroke. It should be noted that the functional neurological prognosis of LAAC in the event of an ischemic stroke event appears to be better, potentially reducing the incidence of disabling/fatal stroke events defined by mRS Score [24]. These findings should be interpreted with caution given the retrospective nature of our study and the heterogeneity in OAC dosing in real-world practice. Moreover, there was no significant difference between the two groups in the occurrence of fatal events, which may be related to the low probability of fatal strokes in the lower-risk group itself coupled with the smaller risk of comorbidities with other lethal diseases, resulting in a lower overall fatality rate in both groups.

In our study, the intervention group incorporated several patients who underwent AF ablation concomitantly to LAAC procedures. Our study results showed that AF ablation did not demonstrate deleterious effects on adverse efficacy events. Consistent with our findings, the OPTION study, with a 36-month follow-up, demonstrated that the timing of LAAC—whether performed concomitantly with or sequentially after ablation—did not significantly affect the rates of primary efficacy events, including all-cause death, stroke, or systemic embolism [25]. Combined with our 99.46% technical success rate, these findings suggest that LAAC can be safely performed alongside AF ablation and that the combined procedures are feasible in terms of long-term TE events and cardiovascular death prevention. The non-inferiority of the incidence of composite efficacy endpoints and major bleeding in the OPTION Clinical Trials of high-risk AF patients can also testify to the above viewpoints. In addition, pulsed field ablation (PFA), a more updated method, was also performed in combination with LAAC in a clinical study, showing that the mean procedure time was reduced by 1/2 compared with combined radiofrequency ablation or cryoablation, and its safety was shown by there being no postoperative safety events within 7 days of intervention [26].

In terms of safety, early safety risks focused on complications related to procedural manipulation and devices (e.g., pericardial effusion, device dislodgement, puncture site hematoma) in the LAAC group, consistent with previous studies [11,27]. Procedure-related pericardial effusion was the most frequent complication in this study. According to the classification [28], two cases occurred intraoperatively, while another two presented with delayed onset (>48 h post-procedure) progressing to cardiac tamponade. One intraoperative case involved significant cardiac perforation with persistent active bleeding, requiring surgical intervention and autologous blood transfusion, which is a rare complication with an estimated incidence of only 0.5%. Among the two cases of delayed pericardial effusion, both showed marked improvement following percutaneous pericardiocentesis, although one patient succumbed to massive bleeding a month post-procedure due to coagulopathy. Device embolization is a serious but infrequent complication. In a retrospective analysis of over 120,000 Watchman device implantations, the in-hospital rate of device embolization was 0.07%, with an additional 0.06% occurrence within 45 days post-discharge. Approximately 14% of patients with in-hospital device embolization died from complications, underscoring the need for vigilance [29]. Notably, previous studies have shown that WATCHMAN 2.5 is less effective at LAA closure than the Amplatzer Amulet LAA occluder and that larger anatomical dimensions usually predicted severe PDL with the Watchman device, possibly resulting in the occurrence of adverse events [30].

Adverse events occurring after 7 days from the initiation of treatment were predominantly bleeding. Notably, there were two cases of clinically significant bleeding in the OAC group (intra-articular and gastrointestinal bleeding, respectively) and one case of major bleeding in the LAAC group (mentioned above), which was not consistent with the previous statement of LAAC reducing the risk of major bleeding compared with OAC [27,31]. The discrepancy between our findings and previous studies on LAAC’s bleeding risk reduction may stem from several factors. Our smaller sample size has limited statistical power to detect significant differences. In addition, bleeding rates may have been influenced by differences in patient selection, with fewer patients at high risk or with contraindications to OACs. The decreased rate of major bleeding events with LAAC may be because LAAC reduces thrombosis by improving blood vertexing and retention within the left atrium and lowering the need for long-term continuous anticoagulation. In addition, anticoagulants have their own safety profiles. Vitamin K antagonists (VKAs) are beneficial for patients with severe renal impairment (eGFR < 30 mL/min) [32]. However, the occurrence of liver dysfunction in four patients on warfarin in this study highlights the systemic adverse effects associated with VKAs. As for DOACs, a meta-analysis that included the four RCTs mentioned above showed that DOACs reduced the risk of intracranial hemorrhage by 50% compared with VKAs [18] but came with an increased risk of gastrointestinal hemorrhage, which is thought to be related to the incomplete absorption of DOACs from the gastrointestinal tract and the dose-dependent relative clotting strength demonstrated by the different types of DOACs on the surface of the local gastrointestinal mucosa [33]. In terms of major bleeding, standard-dose DOACs were also confirmed in a COMBINE AF-based meta-study to outperform warfarin in younger patients regardless of gender, and their performance did not differ from that of warfarin use in older patients [34].

In our study, OACs demonstrated superior efficacy in preventing TE events compared to LAAC in intermediate-to-borderline high-risk NVAF patients, with comparable risks of clinically relevant bleeding, easier management, and fewer procedure-related complications, making them a seemingly more favorable choice. Nevertheless, there are complexities associated with the clinical use of OACs. In addition, despite adherence to oral anticoagulation, 1–2% of AF patients still experience breakthrough strokes, possibly due to inadequate anticoagulation, individual variability, and other thrombogenic factors [35]. Further research is needed to address these challenges.

Study limitations: This study has several limitations that should be acknowledged. First, as a single-center retrospective analysis, our findings should be interpreted as complementary real-world evidence rather than modifying or superseding current guideline recommendations. Second, although propensity score matching was performed to balance baseline covariates, residual imbalances persisted between groups. Future studies should employ more robust statistical methods such as inverse probability weighting (IPW) or doubly robust estimation to better address confounding. Third, the relatively low absolute number of primary efficacy outcome events (e.g., stroke and major bleeding) may have limited the statistical power, potentially affecting the precision and generalizability of our results, particularly in subgroup analyses. Larger prospective studies are warranted to validate these findings and enhance the detection of rare adverse events.

## 5. Conclusions

In this retrospective cohort study, OAC was associated with a significantly lower incidence of the primary composite efficacy endpoint (TIA, systemic embolism, and cardiovascular death) compared to LAAC; however, no statistically significant difference was observed between the two groups regarding ischemic stroke alone. The incidence of non-procedural clinically relevant bleeding (ISTH major bleeding) was similar between treatment groups. Exploratory subgroup analysis demonstrated no significant difference in composite efficacy endpoints between LAAC alone and combined LAAC with AF ablation. These findings suggest that while OAC may be superior to LAAC in reducing thromboembolic events in low-risk NVAF patients, the choice between these strategies should be individualized based on patient characteristics and preferences, particularly given the comparable bleeding risks.

## Figures and Tables

**Table 1:** Baseline characteristics before and after PSM.

	Before PSM				After PSM			
	LAAC (n = 202)	OACs (n = 860)	SMD	*p*	LAAC (n = 184)	OACs (n = 184)	SMD	*p*
Age, n (%)			0.53	<0.01			0.23	0.09
≤64	106 (52.48)	660 (76.74)			91 (49.46)	111 (60.33)		
65–74	85 (42.08)	187 (21.74)			84 (45.65)	68 (36.96)		
≥75	11 (5.45)	13 (1.51)			9 (4.89)	5 (2.72)		
BMI, M (Q_1_, Q_3_)	25.06 (22.68, 27.18)	24.55 (22.51, 26.80)	0.07	0.34	25.18 (22.84, 27.26)	24.27 (22.51, 26.61)	0.13	0.12
Smoking, n (%)			0.11	0.17			0.20	0.06
No	140 (69.31)	552 (64.19)			128 (69.57)	111 (60.33)		
Yes	62 (30.69)	308 (35.81)			56 (30.43)	73 (39.67)		
Drinking, n (%)			0.06	0.46			0.07	0.49
No	144 (71.29)	635 (73.84)			134 (72.83)	128 (69.57)		
Yes	58 (28.71)	225 (26.16)			50 (27.17)	56 (30.43)		
Heart rate, M (Q_1_, Q_3_)	76.00 (69.25, 89.00)	77.00 (67.00, 88.00)	0.09	0.21	76.00 (69.00, 89.25)	75.00 (67.00, 86.25)	0.08	0.24
SBP, M (Q_1_, Q_3_)	126.00 (114.00, 137.00)	125.00 (114.75, 137.00)	0.05	0.69	126.50 (114.00, 136.00)	124.00 (115.00, 136.00)	0.03	0.77
DBP, M (Q_1_, Q_3_)	78.00 (71.25, 86.00)	78.00 (70.00, 86.00)	0.07	0.39	78.00 (70.75, 85.25)	78.00 (69.00, 87.00)	0.01	0.79
EF, M (Q_1_, Q_3_)	61.60 (56.02, 67.38)	63.70 (58.27, 68.60)	0.14	<0.01	61.65 (56.08, 67.12)	62.60 (56.98, 67.12)	0.01	0.52
BNP, n (%)			0.50	<0.01			0.20	0.06
Normal	80 (39.60)	547 (63.60)			70 (38.04)	88 (47.83)		
Abnormal	122 (60.40)	313 (36.40)			114 (61.96)	96 (52.17)		
CHA_2_DS_2_-VA score, mean ± Standard deviation	1.63 ± 0.48	1.32 ± 0.46	0.67	<0.01	1.61 ± 0.49	1.53 ± 0.50	0.17	0.12
HAS-BLED score, n (%)			0.52	<0.01			0.14	0.55
0	30 (14.85)	273 (31.74)			30 (16.30)	40 (21.74)		
1	82 (40.59)	371 (43.14)			75 (40.76)	71 (38.59)		
2	68 (33.66)	188 (21.86)			60 (32.61)	59 (32.07)		
3	21 (10.40)	27 (3.14)			18 (9.78)	14 (7.61)		
4	1 (0.50)	1 (0.12)			1 (0.54)	0 (0.00)		
Comorbidity								
Cardiac insufficiency, n (%)	44 (21.78)	135 (15.70)	0.16	0.04	42 (22.83)	37 (20.11)	0.07	0.53
Hypertension, n (%)	83 (41.09)	316 (36.74)	0.09	0.25	82 (44.57)	72 (39.13)	0.11	0.29
Diabetes mellitus, n (%)	10 (4.95)	48 (5.58)	0.03	0.72	10 (5.43)	10 (5.43)	0.00	1.00
Hx. of stroke/TIA/SE, n (%)	19 (9.41)	8 (0.93)	0.39	<0.01	6 (3.26)	8 (4.35)	0.06	0.59
Vascular disease, n (%)	2 (0.99)	32 (3.72)	0.18	0.05	2 (1.09)	8 (4.35)	0.20	0.05
Drug Usage								
Aspirin, n (%)	4 (1.98)	11 (1.28)	0.06	0.67	4 (2.17)	1 (0.54)	0.14	0.37
Clopidogrel, n (%)	3 (1.49)	11 (1.28)	0.02	1.00	3 (1.63)	5 (2.72)	0.08	0.72
Diuretics, n (%)	24 (11.88)	106 (12.33)	0.01	0.86	21 (11.41)	32 (17.39)	0.17	0.10
Statin, n (%)	91 (45.05)	323 (37.56)	0.15	0.05	83 (45.11)	80 (43.48)	0.03	0.75
RAAS antagonist, n (%)	67 (33.17)	244 (28.37)	0.10	0.18	64 (34.78)	60 (32.61)	0.05	0.66
Beta inhibitor, n (%)	109 (53.96)	504 (58.60)	0.09	0.23	102 (55.43)	112 (60.87)	0.11	0.29
CCB, n (%)	43 (21.29)	138 (16.05)	0.14	0.07	42 (22.83)	32 (17.39)	0.14	0.19
Amiodarone, n (%)	126 (62.38)	679 (78.95)	0.37	<0.01	114 (61.96)	139 (75.54)	0.06	<0.01
Propafenone, n (%)	2 (0.99)	35 (4.07)	0.20	0.03	1 (0.54)	2 (1.09)	0.06	1.00
Digoxin, n (%)	6 (2.97)	29 (3.37)	0.02	0.77	5 (2.72)	10 (5.43)	0.14	0.19

BMI: body mass index; BNP: brain natriuretic peptide; CCB: calcium channel blocker; DBP: diastolic blood pressure; EF: ejection fraction; SBP: systolic blood pressure; SE: systemic embolism; TIA: transient ischemic attack.

**Table 2:** Efficacy and non-procedural endpoints during follow-up.

	LAAC Group (n = 184)			OAC Group (n = 184)				
	No. of Events	Percentage of Patients	Rate/1000 Person-Months	No. of Events	Percentage of Patients	Rate/1000 Person-Months	HR (95% CI)	Log-Rank *p*-Value
Primary efficacy endpoints	20	10.87	219.78	6	3.26	49.18	3.09 (1.22–7.85)	0.01
Thromboembolic events	15	8.15	164.84	4	2.17	32.79	3.61 (1.17–11.11)	0.02
Stroke and TIA	13	7.07	142.86	4	2.17	32.79	2.94 (0.93–9.28)	0.05
All stroke	11	5.98	120.88	4	2.17	32.79	2.49 (0.77–8.07)	0.12
Ischemic stroke	11	5.98	120.88	4	2.17	32.79	2.49 (0.77–8.07)	0.12
Hemorrhagic stroke	0	-	-	0	-	-	-	-
TIA	2	1.09	21.98	0	-	-	-	-
Systemic embolism	2	1.09	21.98	0	-	-	-	-
CV death	5	2.72	54.95	2	1.09	16.39	2.00 (0.37–10.94)	0.41
All-cause death	6	3.26	65.93	4	2.17	32.79	1.27 (0.34–4.71)	0.72
Non-procedural clinical-relevant bleeding	1	0.54	10.99	2	1.09	16.39	0.500 (0.045–5.541)	0.56
Minor bleeding	0	-	-	2	1.09	16.39	-	-
Major bleeding	1	0.54	10.99	0	-	-	-	-

**Table 3:** Univariate and multivariate COX regression analyses on primary efficacy endpoint.

Variables	Univariate Cox Regression		Multivariable Cox Regression	
	*p*	HR (95% CI)	*p*	HR (95% CI)
Therapy				
OACs		1.00 (Reference)		1.00 (Reference)
LAAC	0.02	3.09(1.21~7.84)	0.03	2.87(1.03~7.51)
Age				
<65		1.00 (Reference)		1.00 (Reference)
≥65	0.27	1.54 (0.71~3.34)	0.74	1.18 (0.45~3.12)
CHA_2_DS_2_-VA Score				
1		1.00 (Reference)		1.00 (Reference)
2	0.30	1.52 (0.69~3.36)	0.60	0.76 (0.27~2.14)
HAS-BLED Score				
≤2		1.00 (Reference)		1.00 (Reference)
≥3	<0.01	4.28 (1.71~10.73)	0.02	3.59 (1.24~10.33)
Pattern of AF				
Paroxysmal		1.00 (Reference)		1.00 (Reference)
Persistent	0.12	2.05 (0.82~5.12)	0.90	1.07 (0.39~2.95)
Ejection Fraction	0.02	0.97 (0.94~0.99)	0.04	0.96 (0.93~0.99)
BNP/NT-pro BNP				
Normal		1.00 (Reference)		1.00 (Reference)
Abnormal	<0.01	4.32 (1.48~12.54)	0.06	3.08 (0.97~9.77)

**Table 4:** Treatment information and procedural- and device-related events.

LAAC Relevant Data	No.	Percentage (%)
LAAC device diameter (mm)		
≤30	107	58.15
>30	77	41.85
Success rate of LAAC	183	99.46
Device- or procedural-related events of LAAC		
Device dislodgement	1	0.54
Pericardial effusion	4	2.17
No intervention	1	0.54
Pericardiocentesis required	3	1.53
Groin hematoma	1	0.54
Major bleeding	3	1.53
Procedural-related stroke/transient ischemic attack	0	0.00
Device/Air embolism	1	0.54
Procedural-related death	0	0.00
Nonclinical bleeding during the sequential anticoagulation period	6	0.03
Sequential anticoagulation therapy in 6 weeks after LAAC		
OACs	177	96.19
OACs with single-antiplatelet therapy	7	3.81
Peri-device leakage > 5 mm post-LAAC	2	1.09
OAC group’s relevant data		
Type of OACs		
Warfarin	64	34.78
Direct oral anticoagulants	120	65.22
Side effects of OACs		
Anticoagulant-related bleeding	14	7.60
Clinically relevant	2	1.09
Nonclinically relevant	12	6.52
Severe gastrointestinal symptoms	2	1.09
Drug-related headache/dizziness	2	1.09
Drug-related liver dysfunction	4	2.17

## Data Availability

The raw data supporting the conclusions of this article will be made available by the authors on request.

## Abbreviations

AF	atrial fibrillation.
CTA	CT angiography.
INR	international normalized ratio.
LAAC	left atrial appendage closure.
DOACs	direct oral anticoagulants.
NVAF	non-valvular atrial fibrillation.
OACs	oral anticoagulants.
PSM	propensity score matching.
RCT	randomized controlled clinical trial.
RFCA	radiofrequency catheter ablation.
SE	systemic embolism.
TEE	transesophageal echocardiography.
TE	thromboembolic.
TIA	transient ischemic attack.

## References

[1] Hindricks G., Potpara T., Dagres N., Arbelo E., Bax J.J., Blomström-Lundqvist C., Boriani G., Castella M., Dan G.A., Dilaveris P.E. (2021). 2020 ESC Guidelines for the diagnosis and management of atrial fibrillation developed in collaboration with the European Association for Cardio-Thoracic Surgery (EACTS): The Task Force for the diagnosis and management of atrial fibrillation of the European Society of Cardiology (ESC) Developed with the special contribution of the European Heart Rhythm Association (EHRA) of the ESC. Eur. Heart J..

[2] Cheng S., He J., Han Y., Han S., Li P., Liao H., Guo J. (2024). Global burden of atrial fibrillation/atrial flutter and its attributable risk factors from 1990 to 2021. Europace.

[3] Scheitz J.F., Nolte C.H., Doehner W., Hachinski V., Endres M. (2018). Stroke-heart syndrome: Clinical presentation and underlying mechanisms. Lancet Neurol..

[4] Marini C., De Santis F., Sacco S., Russo T., Olivieri L., Totaro R., Carolei A. (2005). Contribution of atrial fibrillation to incidence and outcome of ischemic stroke: Results from a population-based study. Stroke.

[5] Van Gelder I.C., Rienstra M., Bunting K.V., Casado-Arroyo R., Caso V., Crijns H., De Potter T.J.R., Dwight J., Guasti L., Hanke T. (2024). 2024 ESC Guidelines for the management of atrial fibrillation developed in collaboration with the European Association for Cardio-Thoracic Surgery (EACTS). Eur. Heart J..

[6] Cheng C.Y., Lian T.Y., Zhu X.J., Virdone S., Sun K., Camm J., Li X.M., Goto S., Pieper K., Kayani G. (2025). Atrial fibrillation outcomes in patients from Asia and non-Asia countries: Insights from GARFIELD-AF. Open Heart.

[7] Teppo K., Lip G.Y.H., Airaksinen K.E.J., Halminen O., Haukka J., Putaala J., Mustonen P., Linna M., Hartikainen J., Lehto M. (2024). Comparing CHA(2)DS(2)-VA and CHA(2)DS(2)-VASc scores for stroke risk stratification in patients with atrial fibrillation: A temporal trends analysis from the retrospective Finnish AntiCoagulation in Atrial Fibrillation (FinACAF) cohort. Lancet Reg. Health Eur..

[8] Chan Y.H., Lee H.F., Wang C.L., Chang S.H., Yeh C.H., Chao T.F., Yeh Y.H., Chen S.A., Kuo C.T. (2019). Comparisons of Rivaroxaban Following Different Dosage Criteria (ROCKET AF or J-ROCKET AF Trials) in Asian Patients with Atrial Fibrillation. J. Am. Heart Assoc..

[9] Collado F.M.S., Lama von Buchwald C.M., Anderson C.K., Madan N., Suradi H.S., Huang H.D., Jneid H., Kavinsky C.J. (2021). Left Atrial Appendage Occlusion for Stroke Prevention in Nonvalvular Atrial Fibrillation. J. Am. Heart Assoc..

[10] Belgaid D.R., Khan Z., Zaidi M., Hobbs A. (2016). Prospective randomized evaluation of the watchman left atrial appendage closure device in patients with atrial fibrillation versus long-term warfarin therapy: The PREVAIL trial. Int. J. Cardiol..

[11] Holmes D.R., Reddy V.Y., Turi Z.G., Doshi S.K., Sievert H., Buchbinder M., Mullin C.M., Sick P. (2009). Percutaneous closure of the left atrial appendage versus warfarin therapy for prevention of stroke in patients with atrial fibrillation: A randomised non-inferiority trial. Lancet.

[12] Lip G.Y., Nieuwlaat R., Pisters R., Lane D.A., Crijns H.J. (2010). Refining clinical risk stratification for predicting stroke and thromboembolism in atrial fibrillation using a novel risk factor-based approach: The euro heart survey on atrial fibrillation. Chest.

[13] Carlin S., Cuker A., Gatt A., Gendron N., Hernández-Gea V., Meijer K., Siegal D.M., Stanworth S., Lisman T., Roberts L.N. (2024). Anticoagulation for stroke prevention in atrial fibrillation and treatment of venous thromboembolism and portal vein thrombosis in cirrhosis: Guidance from the SSC of the ISTH. J. Thromb. Haemost..

[14] Rezende S.M., Neumann I., Angchaisuksiri P., Awodu O., Boban A., Cuker A., Curtin J.A., Fijnvandraat K., Gouw S.C., Gualtierotti R. (2024). International Society on Thrombosis and Haemostasis clinical practice guideline for treatment of congenital hemophilia A and B based on the Grading of Recommendations Assessment, Development, and Evaluation methodology. J. Thromb. Haemost..

[15] Zhang Z., Zhou J., Lin Q., Wang C., Huang Y., Dai Y., Zuo W., Liu N., Xiao Y., Liu Q. (2024). Overcoming barriers for left atrial appendage thrombus: A systematic review of left atrial appendage closure. BMC Cardiovasc. Disord..

[16] Joglar J.A., Chung M.K., Armbruster A.L., Benjamin E.J., Chyou J.Y., Cronin E.M., Deswal A., Eckhardt L.L., Goldberger Z.D., Gopinathannair R. (2024). 2023 ACC/AHA/ACCP/HRS Guideline for the Diagnosis and Management of Atrial Fibrillation: A Report of the American College of Cardiology/American Heart Association Joint Committee on Clinical Practice Guidelines. Circulation.

[17] Anjum M., Ariansen I., Hjellvik V., Selmer R., Kjerpeseth L.J., Skovlund E., Myrstad M., Ellekjær H., Christophersen I.E., Tveit A. (2024). Stroke and bleeding risk in atrial fibrillation with CHA2DS2-VASC risk score of one: The Norwegian AFNOR study. Eur. Heart J..

[18] Ruff C.T., Giugliano R.P., Braunwald E., Hoffman E.B., Deenadayalu N., Ezekowitz M.D., Camm A.J., Weitz J.I., Lewis B.S., Parkhomenko A. (2014). Comparison of the efficacy and safety of new oral anticoagulants with warfarin in patients with atrial fibrillation: A meta-analysis of randomised trials. Lancet.

[19] Yamashiro K., Kurita N., Tanaka R., Ueno Y., Miyamoto N., Hira K., Nakajima S., Urabe T., Hattori N. (2019). Adequate Adherence to Direct Oral Anticoagulant is Associated with Reduced Ischemic Stroke Severity in Patients with Atrial Fibrillation. J. Stroke Cerebrovasc. Dis..

[20] Hsu J.C., Maddox T.M., Kennedy K.F., Katz D.F., Marzec L.N., Lubitz S.A., Gehi A.K., Turakhia M.P., Marcus G.M. (2016). Oral Anticoagulant Therapy Prescription in Patients with Atrial Fibrillation Across the Spectrum of Stroke Risk: Insights From the NCDR PINNACLE Registry. JAMA Cardiol..

[21] Connolly S.J., Ezekowitz M.D., Yusuf S., Eikelboom J., Oldgren J., Parekh A., Pogue J., Reilly P.A., Themeles E., Varrone J. (2009). Dabigatran versus warfarin in patients with atrial fibrillation. N. Engl. J. Med..

[22] Patel M.R., Mahaffey K.W., Garg J., Pan G., Singer D.E., Hacke W., Breithardt G., Halperin J.L., Hankey G.J., Piccini J.P. (2011). Rivaroxaban versus warfarin in nonvalvular atrial fibrillation. N. Engl. J. Med..

[23] Giugliano R.P., Ruff C.T., Braunwald E., Murphy S.A., Wiviott S.D., Halperin J.L., Waldo A.L., Ezekowitz M.D., Weitz J.I., Špinar J. (2013). Edoxaban versus warfarin in patients with atrial fibrillation. N. Engl. J. Med..

[24] Sacks D., Baxter B., Campbell B.C.V., Carpenter J.S., Cognard C., Dippel D., Eesa M., Fischer U., Hausegger K., Hirsch J.A. (2018). Multisociety Consensus Quality Improvement Revised Consensus Statement for Endovascular Therapy of Acute Ischemic Stroke. Int. J. Stroke.

[25] Saliba W., Nair D., Swarup V., Hall T., Iyer V., Pérez G.C., Weiner S., Shah M., Islam N., Grygier M. (2025). Comparison of left atrial appendage closure and oral anti-coagulation after catheter ablation for atrial fibrillation: Concomitant and sequential cohorts of the OPTION randomized controlled trial. Heart Rhythm..

[26] Beney J., Galea R., Siontis G., Gräni C., Kueffer T., Brugger N., Reichlin T., Räber L., Roten L. (2024). Feasibility study on atrial fibrillation ablation with pulsed field ablation and concomitant occlusion of the left atrial appendage. Europace.

[27] Osmancik P., Herman D., Neuzil P., Hala P., Taborsky M., Kala P., Poloczek M., Stasek J., Haman L., Branny M. (2022). 4-Year Outcomes After Left Atrial Appendage Closure Versus Nonwarfarin Oral Anticoagulation for Atrial Fibrillation. J. Am. Coll. Cardiol..

[28] Tzikas A., Holmes D.R., Gafoor S., Ruiz C.E., Blomström-Lundqvist C., Diener H.C., Cappato R., Kar S., Lee R.J., Byrne R.A. (2017). Percutaneous left atrial appendage occlusion: The Munich consensus document on definitions, endpoints, and data collection requirements for clinical studies. Europace.

[29] Friedman D.J., Freeman J.V., Zimmerman S., Tan Z., Pereira L., Faridi K.F., Curtis J.P. (2023). Watchman device migration and embolization: A report from the NCDR LAAO Registry. J. Cardiovasc. Electrophysiol..

[30] Lakkireddy D., Nielsen-Kudsk J.E., Windecker S., Thaler D., Price M.J., Gambhir A., Gupta N., Koulogiannis K., Marcoff L., Mediratta A. (2023). Mechanisms, predictors, and evolution of severe peri-device leaks with two different left atrial appendage occluders. Europace.

[31] Magnocavallo M., Della Rocca D.G., Vetta G., Mohanty S., Gianni C., Polselli M., Rossi P., Parlavecchio A., Fazia M.V., Guarracini F. (2024). Lower rate of major bleeding in very high risk patients undergoing left atrial appendage occlusion: A propensity score-matched comparison with direct oral anticoagulant. Heart Rhythm..

[32] Chiang C.E., Chao T.F., Choi E.K., Lim T.W., Krittayaphong R., Li M., Chen M., Guo Y., Okumura K., Lip G.Y.H. (2022). Stroke Prevention in Atrial Fibrillation: A Scientific Statement of JACC: Asia (Part 2). JACC Asia.

[33] Vanassche T., Hirsh J., Eikelboom J.W., Ginsberg J.S. (2014). Organ-specific bleeding patterns of anticoagulant therapy: Lessons from clinical trials. Thromb. Haemost..

[34] Carnicelli A.P., Hong H., Connolly S.J., Eikelboom J., Giugliano R.P., Morrow D.A., Patel M.R., Wallentin L., Alexander J.H., Cecilia Bahit M. (2022). Direct Oral Anticoagulants Versus Warfarin in Patients with Atrial Fibrillation: Patient-Level Network Meta-Analyses of Randomized Clinical Trials with Interaction Testing by Age and Sex. Circulation.

[35] Galea R., Seiffge D., Räber L. (2023). Atrial Fibrillation and Ischemic Stroke despite Oral Anticoagulation. J. Clin. Med..

